# Oral liposomal iron vs. oral iron polymaltose in children with chronic kidney disease iron deficiency anemia: a cross-over study

**DOI:** 10.1007/s00467-025-07138-w

**Published:** 2026-01-15

**Authors:** Happy Sawires, Eman Abobakr Abd Alazem, Fatma Atia, Amr Salem, Amira Samy, Mohamed Gamal

**Affiliations:** Cairo University, Cairo, Egypt

**Keywords:** Pediatric, CKD, Iron deficiency anemia, Iron therapy, Iron polymaltose complex, Liposomal iron

## Abstract

**Background:**

Limited data exist on the use of novel iron therapies in children with chronic kidney disease (CKD). We conducted a cross-over study to compare iron polymaltose complex (IPC) and liposomal iron in pediatric patients with CKD and iron deficiency anemia (IDA).

**Methods:**

Cross-over study of 33 children with CKD and IDA was conducted. They were randomized into 2 groups (group A: 17 patients, group B: 16 patients) to receive either liposomal iron or IPC for 3 months. After an 8-week washout period, they were switched to the other therapy. Red cell and iron indices, as well as bone minerals and 25(OH)D_3_, were measured at baseline and after each 3-month period. A follow-up visit was conducted at 4 weeks during the treatment period to report any possible adverse events.

**Results:**

Hb levels increased by at least 1 g/dL in 48% following liposomal iron therapy and 51.5% following IPC therapy. There was no statistically significant difference in ΔHb, ΔFe, ΔsTR (transferrin receptor), or ΔTSAT (transferrin saturation) levels between the groups (*p* > 0.05). By mixed model analysis, IPC showed a higher Hb and TSAT and lower TRresponse compared with liposomal iron. IPC, but not liposomal iron, led to a significant reduction in serum phosphorus in both groups. Thirty-six percent of IPC recipients experienced adverse effects, compared to 3% of liposomal iron recipients.

**Conclusions:**

Both IPC and liposomal iron effectively improved iron status in children with CKD and IDA. However, IPC indicated a superior response, whereas liposomal iron was associated with a more favorable tolerability profile.

**Graphical abstract:**

**Figure:**
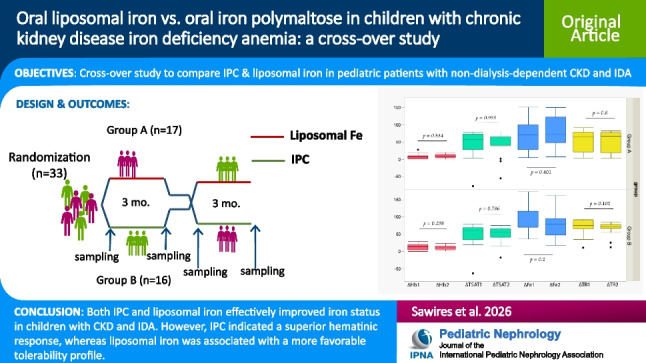
A higher resolution version of the Graphical abstract is available as Supplementary information

**Supplementary Information:**

The online version contains supplementary material available at 10.1007/s00467-025-07138-w.

## Introduction

Iron deficiency anemia (IDA) is one of the most frequent complications in children with chronic kidney disease (CKD), even in early stages. Multiple factors contribute to the development of IDA in this population, including reduced dietary iron intake, chronic blood loss, impaired intestinal absorption, and functional iron deficiency secondary to inflammation-mediated upregulation of hepcidin [1].

Oral iron supplementation is recommended for iron deficiency in non-dialysis dependent CKD (NDD-CKD) because it carries a lower risk of hypersensitivity reactions and is more cost-effective compared to intravenous treatment [2]. However, traditional oral iron salts have limited bioavailability, and high doses may hinder absorption by increasing hepcidin levels [3]. Moreover, inflammation in CKD leads to elevated serum ferritin levels, which may not accurately reflect iron status. This makes functional iron deficiency more common and highlights the need for formulations that can be effectively absorbed even under inflammatory conditions [4].

Liposomal iron has emerged as a promising formulation with improved tolerance and enhanced bioavailability, bypassing the hepcidin–ferroportin blockade [5]. Several studies in adult and pediatric CKD populations have demonstrated that liposomal iron effectively increases hemoglobin and iron indices with fewer side effects, and may achieve comparable hemoglobin responses to intravenous iron over time [6–9].

Iron polymaltose complex (IPC), though widely used in children for IDA, has shown a slower hemoglobin response compared to ferrous salts but with good tolerability [10]. However, data on IPC use in CKD patients are limited. This gap highlights the need for comparative studies evaluating these two oral iron formulations in CKD, particularly in children. Therefore, we conducted this cross-over study to compare IPC and liposomal iron in pediatric patients with NDD-CKD and iron deficiency anemia (IDA).

## Methods

A total of 33 patients aged between 1 and 15 years of both genders with CKD were enrolled in a prospective two-period crossover study. Eligible participants included stratified random sample of children with CKD stages 2 to 5 who were not receiving kidney replacement therapy (KRT). Anemia was defined as hemoglobin below the lower limit of normal for age and IDA was diagnosed when transferrin saturation <20% and serum ferritin < 100 ng/ml [11]. The CKD diagnostic criteria were based on the guidelines proposed by Kidney Disease: Improving Global Outcomes (KDIGO) CKD Work Group [12]. The recruitment criteria were as follows: no history of iron supplements consumption within the past 3 months and no history of blood transfusion within the past 4 months. ESA treatment was not used in any patient during the course of the study. We excluded children with poor adherence to the medications and follow-up (*N* = 4), hematological diseases, immune disorder affecting hematological system, born prematurely or with low birth weight. Patients were recruited from the Pediatric Nephrology Outpatient Clinic at Cairo University Children’s Hospital. The study was conducted in accordance with the ethical principles outlined in the Declaration of Helsinki. Written informed consent was obtained from the caregivers of all participants, and the study received approval from the institutional ethics committee. The Research Ethical Committee of the Faculty of Medicine of Cairo University approved and monitored this study (N-87–2023).

The patients were randomized into 2 groups: A (17 patients) and B (16 patients). Demographic data were collected, and baseline blood samples were obtained for measurement of complete blood count (CBC), serum Na, K, Ca, P, ALP, 25 (OH) D_3_, iron, ferritin, total iron binding capacity (TIBC) and soluble transferrin receptors (sTR). Transferrin saturation (TSAT) was calculated [(serum Fe/TIBC) × 100].

Patients in Group A received oral liposomal iron (Fortiferrum® 240 mg liposomal Fe, Splendid Pharmaceuticals) at a dose of 1.4 mg/kg/day administered once daily, while those in Group B were given oral iron polymaltose complex (IPC) (Enrich® equivalent to elemental iron 50 mg/ml, Marcyrl Pharmaceuticals) at a dose of 6 mg/kg, divided into two doses and taken on an empty stomach for a duration of 3 months. A follow-up visit was conducted at four weeks during the treatment period to report any possible adverse events (caregivers completed a questionnaire to report and grade (mild–moderate–severe) various new symptoms not attributable to CKD itself or other illnesses, including gastrointestinal symptoms (metallic taste, nausea, vomiting, heartburn, abdominal pain, bloating, constipation, diarrhea), immune reactions, dermatological manifestations (pruritus, rash, urticaria, erythema), and any other reported symptoms) and to assess medication adherence (caregiver report with number of sachets and bottles). Patients who developed moderate or severe adverse effects during follow-up were instructed to discontinue the medication and switch to an alternative therapy. At the end of the three-month period, patients were evaluated for potential adverse effects, and blood samples were collected to reassess the same laboratory parameters measured at baseline.

**Fig. 1:**
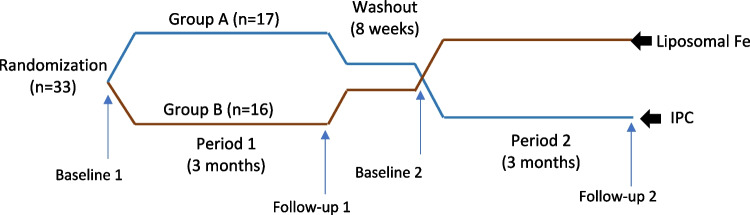
Study design

Patients in both groups were instructed to discontinue iron supplementation for an 8-week washout period. Following this interval, a second set of baseline blood samples were collected to reassess the same laboratory parameters. Subsequently, treatment regimens were crossed over: patients in Group A received IPC, while those in Group B were administered liposomal iron for an additional 3 months. At the end of the study period, patients were evaluated for potential adverse effects, and blood samples were collected once more to evaluate the same baseline parameters (Fig. 1). Although the protocol allowed for discontinuation in the event of moderate or severe adverse effects, no patient met these criteria during the treatment phase.

The primary outcome was the efficacy of the two iron preparations after 3 months, evaluated through the percentage of increase in Hb (ΔHb), Fe (ΔFe), and TSAT (ΔTSAT) and the percentage of decrease of sTR (ΔsTR). The secondary outcomes focused on safety, including the occurrence of any adverse events related to the medications.

### Statistical analysis

Quantitative data were presented as mean and standard deviation. Qualitative (Categorical) data were presented as frequencies and percentages. The percentage increase in Hb (ΔHb), serum Fe levels (ΔFe), and TSAT (ΔTSAT) were calculated using the following formula: [(post-treatment value − pre-treatment value)/post-treatment value] × 100. Specifically, for hemoglobin and serum iron: [(Hbafter − Hbbefore)/Hbafter] × 100 and [(Feafter − Febefore)/Feafter] × 100, respectively. The percentage decrease in soluble transferrin receptor (sTR) levels (ΔsTR) was calculated as follows: [(sTRbefore − sTRafter)/sTRbefore] × 100.

Paired T test was used to compare paired parametric variables while Wilcoxon test was used for paired non-parametric variables. A linear mixed-effects model was fitted. Fixed effects included treatment, period, sequence, and an optional carryover term. Subject (nested within sequence) was modeled as a random effect. This structure accounts for the two-period crossover design and the between-subject variability.

The significance level was set at *p* < 0.05. Statistical analysis was performed with SPSS 29.0 (statistical package for scientific studies) for Macintosh.

## Results

A total of 33 patients completed the study and were divided into two groups: Group A (17 patients) and Group B (16 patients). In Group A, the underlying diagnosis of CKD was ascertainable in 15 patients (88%), comprising 7 patients with obstructive uropathy, 6 patients with inherited nephropathy, and 2 patients with glomerulopathies. Conversely, in Group B, the underlying diagnosis was known in 14 patients (87.5%), including 6 patients with obstructive uropathy, 7 patients with inherited nephropathy, and 1 patient with glomerulopathy. Of the 33 patients in our cohort, 17 were newly diagnosed, whereas the other 20 had an established diagnosis and were already being followed. The demographic data of the patients is presented in Table 1. The markedly low baseline hematologic indices likely reflect the cohort’s history of repeated courses of oral iron therapy (ferrous sulfate, gluconate, or fumarate), which had produced consistently inadequate hematologic responses.

**Table 1:** Demographic characteristics of studied participants

	Group A (N = 17)	Group B (N = 16)	*p* value
Age (years)	7.12 ± 3.65	6.94 ± 2.81	0.082
Gender			
Male (%)	11 (65%)	12 (75%)	0.708
Female (%)	6 (35%)	4 (25%)	
Weight-for-age z-score	–0.52 ± 1.46	–1.14 ± 0.92	0.220
Median (IQR)	–0.82 (–1.6, 0.4)	–1.08 (–1.6, –0.57)	
Height-for-age z score	–0.47 ± 0.88	–0.56 ± 1.09	0.652
Median (IQR)	–0.75 (–1.04, 0.35)	–0.75 (–1.2, 0.07)	
S. creatinine (mg/dL)	2.61 ± 0.58	2.65 ± 0.66	0.836
CKD staging			
Stage 2 (%)	1 (5.8%)	0 (0%)	
Stage 3 (%)	9 (53%)	7 (43.8%)	0.359
Stage 4 (%)	7 (41.2%)	7 (43.8%)	
Stage 5(%)	0 (0%)	2 (12.4%)	
S. calcium (mg/dL)	9.64 ± 0.73	9.68 ± 0.69	0.855
S. phosphorus (mg/dL)	5.07 ± 1.12	4.81 ± 0.91	0.475
ALP (IU/L)	373.1 ± 215.55	295.81 ± 113.78	0.368
Median (IQR)	320.0 (201, 537)	290.0 (179, 399)	
25 (OH)D_3_(ng/ml)	29.3 ± 3.4	29.7 ± 4.01	0.624
S. sodium (mEq/L)	138.8 ± 1.86	138.15 ± 2.77	0.540
S. potassium (mEq/L)	4.47 ± 0.76	4.68 ± 0.54	0.385
RBCs (×10^6^/mL)	4.03 ± 0.15	4.18 ± 0.14	0.068
Hb (g/dL)	9.21 ± 0.45	8.90 ± 0.6	0.097
HCT (%)	28.22 ± 1.12	27.78 ± 1.9	0.425
MCV (fL)	69.68 ± 3.42	66.83 ± 5.58	0.084
MCH (µg/dL)	22.34 ± 1.08	22.02 ± 1.24	0.424
S. Fe (µg/dL)	35.71 ± 4.91	29.06 ± 7.91	0.074
TIBC (µg/dL)	308.88 ± 57.23	361.6 ± 22.65	0.081
TSAT (%)	11.94 ± 2.98	14.12 ± 4.04	0.086
S. ferritin (ng/ml)	75.78 ± 20.89	71.42 ± 23.57	0.577
sTR (ng/ml)	9862.37 ± 4783.9	9224.8 ± 2929.4	0.650
	Median = 9370 IQ (4616, 14250)	Median = 8540 IQ (6883, 12149)	

*ALP* alkaline phosphatase, *Fe* iron, *Hb* hemoglobin, *HCT* hematocrit, *MCH* mean corpuscular hemoglobin, *MCV* mean corpuscular volume, *TIBC* total iron binding capacity, *sTR* soluble transferrin receptor, *TSAT* transferrin saturation

In our study, hemoglobin levels increased by at least 1 g/dL in 16 patients (48%) following liposomal iron treatment and in 17 patients (51.5%) following IPC therapy. Serum ferritin levels were significantly elevated following IPC and liposomal iron administration (*p* < 0.001 and 0.003, respectively) in group B, while only liposomal iron administration (*p* < 0.001) resulted in an increase in group A.

The red cell indices and iron profile for both patient groups are presented in Tables 2 and 3 (figures are available as online supplementary material).

**Table 2:** Follow-up parameters in Group A

	Liposomal iron			IPC		
	Baseline	After 3 mo	*p* value	Baseline	After 3 mo	*p* value
RBCs (×10^6^/mL)	4.03 ± 0.15	4.39 ± 0.58	0.048	4.16 ± 0.27	4.39 ± 0.52	0.057
Hb (g/dL)	9.21 ± 0.45	9.94 ± 0.94	<0.001	9.43 ± 0.66	10.32 ± 1.02	<0.001
HCT (%)	28.22 ± 1.12	30.55 ± 2.77	0.015	29.08 ± 2.8	31.18 ± 2.91	<0.001
MCV (fL)	69.68 ± 3.42	76.68 ± 4.13	<0.0010	72.06 ± 2.37	77.12 ± 4.6	<0.001
MCH (µg/dL)	22.34 ± 1.08	24.50 ± 1.55	.009	23.42 ± 1.5	25.72 ± 4.0	0.037
S. Fe (µg/dL)	35.71 ± 4.91	59.50 ± 13.84	<0.001	37.47 ± 6.58	62.61 ± 18.71	<0.001
TIBC (µg/dL)	308.88 ± 57.23	244.3 ± 83.10	0.016	322.0 ± 60.07	248.3 ± 49.41	0.007
TSAT (%)	11.94 ± 2.98	27.94 ± 11.96	<0.001	11.94 ± 2.90	26.76 ± 11.57	<0.001
S. ferritin (ng/ml)	75.78 ± 20.89	180.8 ± 90.84	<0.001	121.8 ± 45.26	141.2 ± 48.5	0.193
sTR (ng/ml)	9862.37 ± 4783.9	4867.2 ± 4300	<0.001	7460.2 ± 4054.3	3713.4 ± 3153.3	<0.001
	Median = 9370 IQ (4616, 14250)	Median= 3949 IQ (1138, 7325)		Median = 5880 IQ (3778, 11210)	Median = 3610 IQ (1001, 5741)	
Serum Ca (mg/dL)	9.64 ± 0.73		0.432	9.87 ± 1.89	9.94 ± 1.12	0.129
Serum P (mg/dL)	5.07 ± 1.12	9.81±0.71	0.071	5.12 ± 1.17	4.61 ± 1.21	0.043
25 (OH)D_3_(ng/ml)	29.3 ± 3.4	5.31 ± 0.83	0.117	29.01 ± 3.52	29.86 ± 3.92	0.301
		30.4 ± 4.9				

*ALP* alkaline phosphatase, *Fe* iron, *Hb* hemoglobin, *HCT* hematocrit, *MCH* mean corpuscular hemoglobin, *MCV* mean corpuscular volume, *TIBC* total iron binding capacity, *sTR* soluble transferrin receptor, *TSAT* transferrin saturation

**Table 3:** Follow-up parameters in Group B

	IPC			Liposomal iron		
	Baseline	After 3 mo	*p* value	Baseline	After 3 mo	*p* value
RBCs (×10^6^/mL)	4.18 ± 0.14	4.41 ± 0.44	0.021	4.17 ± 0.31	4.62 ± 0.28	0.002
Hb (g/dL)	8.90 ± 0.6	9.80 ± 0.96	<0.001	9.31 ± 0.76	10.36 ± 0.99	<0.001
HCT (%)	27.78 ± 1.9	30.63 ± 2.19	<0.001	28.77 ± 1.90	32.08 ± 2.93	<0.001
MCV (fL)	66.83 ± 5.58	75.92 ± 4.90	<0.001	67.71 ± 6.18	76.97 ± 3.88	<0.001
MCH (µg/dL)	22.02 ± 1.24	25.65 ± 1.39	0.012	23.46 ± 1.17	25.58 ± 1.69	0.024
S. Fe (µg/dL)	29.06 ± 7.91	61.01 ± 13.51	<0.001	38.80 ± 5.52	65.65 ± 15.15	<0.001
TIBC (µg/dL)	361.6 ± 22.65	225.6 ± 66.91	<0.001	318.3 ± 58.91	243.2.2 ± 66.1	0.003
TSAT (%)	14.12 ± 4.04	30.62 ± 7.51	<0.001	12.7 ± 3.39	28.37 ± 10.13	<0.001
S. ferritin (ng/ml)	71.42 ± 23.57	149.22 ± 38.6	<0.001	115.06 ± 26.47	142.6 ± 38.46	0.003
sTR (ng/ml)	9224.8 ± 2929.4	2530.7 ± 2587.5	<0.001	5461.0 ± 2185.5	2009.7 ± 1816.4	<0.001
	Median = 8540 IQ (6883, 12149)	Median = 1740 IQ (4036, 5681)		Median = 4887.8 IQ (4036, 5681)	Median = 1305 IQ (861.3, 2259.2)	
Serum Ca (mg/dL)	9.68 ± 0.69	9.67 ± 1.94	0.371	9.70 ± 1.96	9.84 ± 2.17	0.211
Serum P (mg/dL)	4.81 ± 0.91	4.63 ± 1.06	0.044	5.12 ± 1.62	5.01 ± 1.21	0.317
25 (OH)D_3_(ng/ml)	29.7 ± 4.01	30.11 ± 4.02	0.281	29.54 ± 4.09	30.42 ± 3.36	0.132

*ALP* alkaline phosphatase, *Fe* iron, *Hb* hemoglobin, *HCT* hematocrit, *MCH* mean corpuscular hemoglobin, *MCV* mean corpuscular volume, *TIBC* total iron binding capacity, *sTR* soluble transferrin receptor, *TSAT* transferrin saturation

**Fig. 2:**
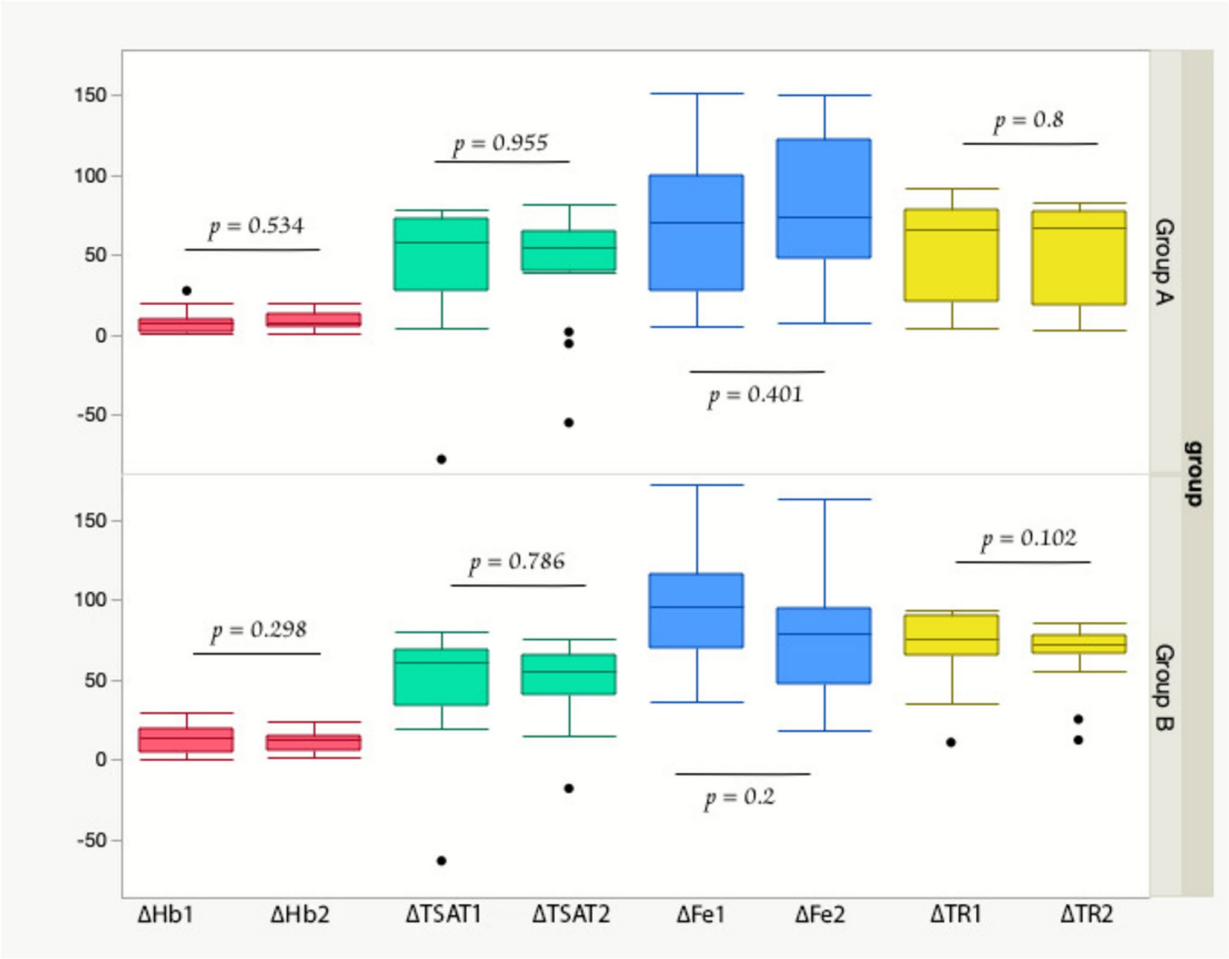
Boxplots of means of ΔHb, ΔTSAT, ΔFe, and ΔsTR in both groups

In group A, there was no statistically significant difference between liposomal iron and IPC in ΔHb, ΔFe, ΔsTR, and ΔTSAT (*p* = 0.534, 0.401, 0.80, and 0.955, respectively). Similarly, in group B, there was no statistically significant difference in ΔHb, ΔFe, ΔsTR, or ΔTSAT (*p* = 0.298, 0.20, 0.102, and 0.786, respectively), Fig. 2.

The mixed model suggests IPC therapy produced significantly higher Hb and TSAT and lower sTR compared with liposomal iron (*p* ≈ 0.039, 0.023, and 0.044 respectively), while the Fe response was not significantly different. Period and carryover effects were not significant, suggesting no strong evidence of residual (washout) effects (Table 4).

**Table 4:** Mixed-effect model analysis

	TSAT		Hb		Fe		TR	
	Estimate (SE)	*p* value	Estimate (SE)	*p* value	Estimate (SE)	*p* value	Estimate (SE)	*p* value
Intercept	96.61 (20.71)	<0.001	21.93 (6.63)	0.002	116.7 939.64)	0.005	115.65 (27.4)	<0.001
Period	–37.94 (19.98)	0.066	–11.14 (6.35)	0.088	–35.35 (38.11)	0.363	–52.9 (26.8)	0.056
Treatment	–8.69 (3.70)	0.023	–2.58 (1.23)	0.039	–11.12 (7.21)	0.129	–9.58 (4.58)	0.044
Carryover	20.06 (13.12)	0.136	7.13 (4.14)	0.095	21.64 (24.94)	0.392	33.09 (17.8)	0.072
Sequence	–2.45 (12.86)	0.864	–1.92 (2.23)	0.547	1.88 (25.06)	0.944	–8.15 (2.13)	0.137

Period (1st vs. 2nd), Treatment (liposomal iron vs. IPC), Sequence (liposomal iron-IPC vs. IPC-liposomal iron)

*Hb* hemoglobin, *Fe* iron, *SE* standard error, *TR*  transferrin receptor, *TSAT* transferrin saturation

IPC exhibited a substantial reduction in serum phosphorus levels in both groups A and B (*p* = 0.043 and 0.044, respectively), while there was a non-significant decrease in both serum calcium and 25(OH)D3 levels (*p* > 0.05). Notably, liposomal iron did not induce any significant changes in these parameters (Tables 2 and 3).

No participants had to stop the iron therapy because of adverse effects. In general, adverse effects were mild and more prevalent with IPC compared to liposomal iron. Specifically, 12 (36%) of IPC recipients experienced adverse effects, whereas only 3 (9%) of liposomal iron recipients experienced adverse effects. This difference was statistically significant (*p* < 0.001).

The adverse effects experienced with IPC included metallic taste (6, 18%), constipation (5, 15%), heartburn (3, 15%), abdominal pain (3, 15%), and vomiting (1, 3%). In contrast, the adverse effects experienced with liposomal iron were limited to heartburn (2, 6%) and abdominal pain (1, 3%).

## Discussion

There is a scarcity of comparative data examining the efficacy of the newer oral iron formulations in children with CKD without KRT and IDA. In this cross-over study, we observed comparable beneficial effects of both IPC and liposomal iron as evidenced by changes in ΔHb, ΔFe, ΔTSAT, and ΔsTR and in terms of increasing red cell indices (Hb, MCV, and MCH). However, the mixed model analysis indicated a superior response of IPC, evidenced by higher Hb and TSAT and lower sTR compared with liposomal iron. Both treatments achieved a comparable proportion of patients with a hemoglobin increase of at least 1 g/dL, while the target TSAT level (>25%) was attained in all participants irrespective of the formulation used. It is worth noting that the KDIGO guidelines and related reviews define iron responsiveness in CKD anemia as an Hb rise of ≥1 g/dL and often targeting TSAT > 20–30% post-therapy [13, 14].

IPC is a ferric complex with maltol that enables soluble iron delivery at a neutral pH. Ferric maltol facilitates iron uptake by enterocytes while keeping the unabsorbed fraction chelated in a redox-inert form, thereby minimizing oxidative stress and gastrointestinal irritation. Before absorption, iron dissociates from the maltol complex, whereas free maltol is independently absorbed, metabolized, and excreted in the urine [15]. Although previous studies have reported comparable effects of IPC and other novel formulations such as liposomal or sucrosomial iron in improving hemoglobin and serum ferritin levels [6, 16], none of these investigations have been conducted in populations with chronic diseases such as CKD.

Contrary to the common belief that oral iron preparations are less effective compared with IV iron due to reduced iron absorption in the gut, substantial evidence from multiple large randomized controlled trials indicates that oral iron can significantly improve iron stores and hemoglobin levels in patients with CKD without KRT [17]. Oral iron in patients with CKD without the use of ESAs typically results in a modest or limited increase in Hb levels. For example, in the FIND-CKD trial, 32.1% of adult patients receiving oral iron alone achieved Hb rise ≥ 1 g/dL within 48 weeks [18].

The response to iron therapy in children with CKD-related anemia is less robust compared to that seen in children with nutritional IDA. In a randomized clinical trial, young children with nutritional IDA experienced a 4.0 g/dL increase in Hb after 3 months of a lower dose of elemental iron daily [19].

Another study reviewing oral Fe therapy in pediatric patients with CKD found that after about three months of treatment, hemoglobin increased modestly from 10.2 to 10.8 g/dL, while transferrin saturation improved from 16% to 21.4%. However, only about 29% of children achieved resolution of anemia by KDIGO criteria, and 35% showed no improvement in anemia at all [20]. Although both IPC and liposomal iron effectively improved iron parameters in our patients, they are not sufficient as standalone therapies for anemia in children with CKD, given the condition’s multifactorial nature.

Despite improvements in iron indices, only about half of the patients achieved a ≥1 g/dL rise in hemoglobin, likely reflecting the altered iron metabolism characteristic of CKD, including elevated hepcidin levels and erythropoietin resistance [21].

In our study, while oral iron therapies can improve anemia parameters in children with CKD, serum ferritin levels often exhibit inconsistent responses. For instance, a randomized double-blind clinical trial in adults with NDD-CKD and IDA demonstrated a significant increase in serum ferritin (95% CI, 144.9 to 195.7 ng/mL; *p* < 0.001) [22]. In contrast, a study involving children with CKD stages II–IV who received oral iron therapy for approximately 3 months found no significant change in ferritin levels (from 55.0 to 44.9 ng/mL), despite improvements in other red cell and iron indices [20]. The absence of a notable ferritin increase in pediatric CKD-related anemia may be attributed to several factors, including altered iron distribution, elevated hepcidin levels, and the chronic inflammatory state associated with CKD [1]. Serum ferritin itself alone is not a diagnostic marker of iron deficiency in inflammatory conditions like CKD [4, 23].

Liposomal iron is an advanced delivery system in which micronized ferric sulfate particles are encapsulated within phospholipid vesicles, enhancing iron transport across cell membranes [24]. In a recent pilot study, liposomal iron administration did not yield substantial effects on Hb levels or the necessity for ESA. However, it did result in a notable elevation of serum iron levels and a concomitant decrease in serum transferrin levels. This effect manifested in a significant enhancement of TSAT [8]. An Italian study involving patients with NDD-CKD and IDA compared liposomal iron to IV iron therapy. After 3 months of liposomal iron treatment, the authors observed a significant average increase in hemoglobin of 0.6 g/dL, with no corresponding change in ferritin levels [7].

Encapsulating iron within a liposomal outer shell helps protect intestinal cells by preventing direct contact between iron and the intestinal mucosa, thereby reducing gastrointestinal side effects. This is particularly important, as the occurrence of adverse reactions such as diarrhea, constipation, and dyspepsia can significantly impact adherence to oral iron therapy [9]. We observed a substantial decrease in the incidence of adverse effects in patients receiving liposomal iron compared to IPC.

Although no significant changes were observed in serum calcium or 25(OH)D₃ levels following administration of either IPC or liposomal iron, a notable reduction in serum phosphorus was seen in patients who received IPC, but not in those given liposomal iron. Not all iron preparations have the same effect on serum phosphorus. A recent meta-analysis demonstrated that oral iron formulations significantly reduced circulating c-terminal FGF23 levels in treated patients, whereas intravenous iron showed no significant effect compared to controls [25]. It has been hypothesized that the carbohydrate components specific to intravenous iron preparations may transiently increase intact FGF23 (iFGF23) levels in osteocytes by reducing the potential for FGF23 cleavage, which is associated with renal phosphate wasting, decreased serum phosphorus, and lower calcitriol levels [26, 27]. Whether this mechanism explains the hypophosphatemic effect of IPC in our study remains to be clarified.

Although this is the first cross-over study that compares novel oral iron preparations for the treatment of IDA in children with CKD, there are some limitations. Firstly, the small sample size limits the generalizability of the findings, although it can be considered a pilot comparison. Secondly, a 3-month treatment period may be insufficient to fully assess long-term efficacy, particularly in a chronic condition like CKD. Thirdly, the lack of blinding may introduce performance or reporting bias. Fourthly, the absence of measurement of PTH and iFGF-23 restricts the conclusions drawn from such therapies regarding bone mineral metabolism. Lastly, the choice of IPC as the comparator with liposomal iron reflects local clinical practice, where this formulation is preferred over ferrous sulfate because of better tolerability and adherence. Future studies comparing both formulations with ferrous sulfate would further clarify their relative efficacy.

In conclusion, both IPC and liposomal iron effectively improved iron status in children with CKD and IDA. However, IPC indicated a superior response as evidenced by higher Hb and TSAT and lower sTR compared with liposomal iron, whereas liposomal iron was associated with a more favorable tolerability profile.

## Supplementary Information

Below is the link to the electronic supplementary material.

Graphical abstract (PPTX 112 KB)

Supplementary file 1 (PNG 21.2 KB )

Supplementary file 2 (PNG 26.0 KB)

## Data Availability

The datasets generated during and/or analysed during the current study are available from the corresponding author on reasonable request.
