# Supplementary material for: Oral liposomal iron vs. oral iron polymaltose in children with chronic kidney disease iron deficiency anemia: a cross-over study
Source: Pediatr Nephrol. 2026 Jan 15;41(6):1803–10. doi: 10.1007/s00467-025-07138-w (PMC13139247; doi:10.1007/s00467-025-07138-w)
Supplement: Supplementary file 1 — Graphical abstract (PPTX 112 KB) [file 467_2025_7138_MOESM1_ESM.pptx]

## Slide 1
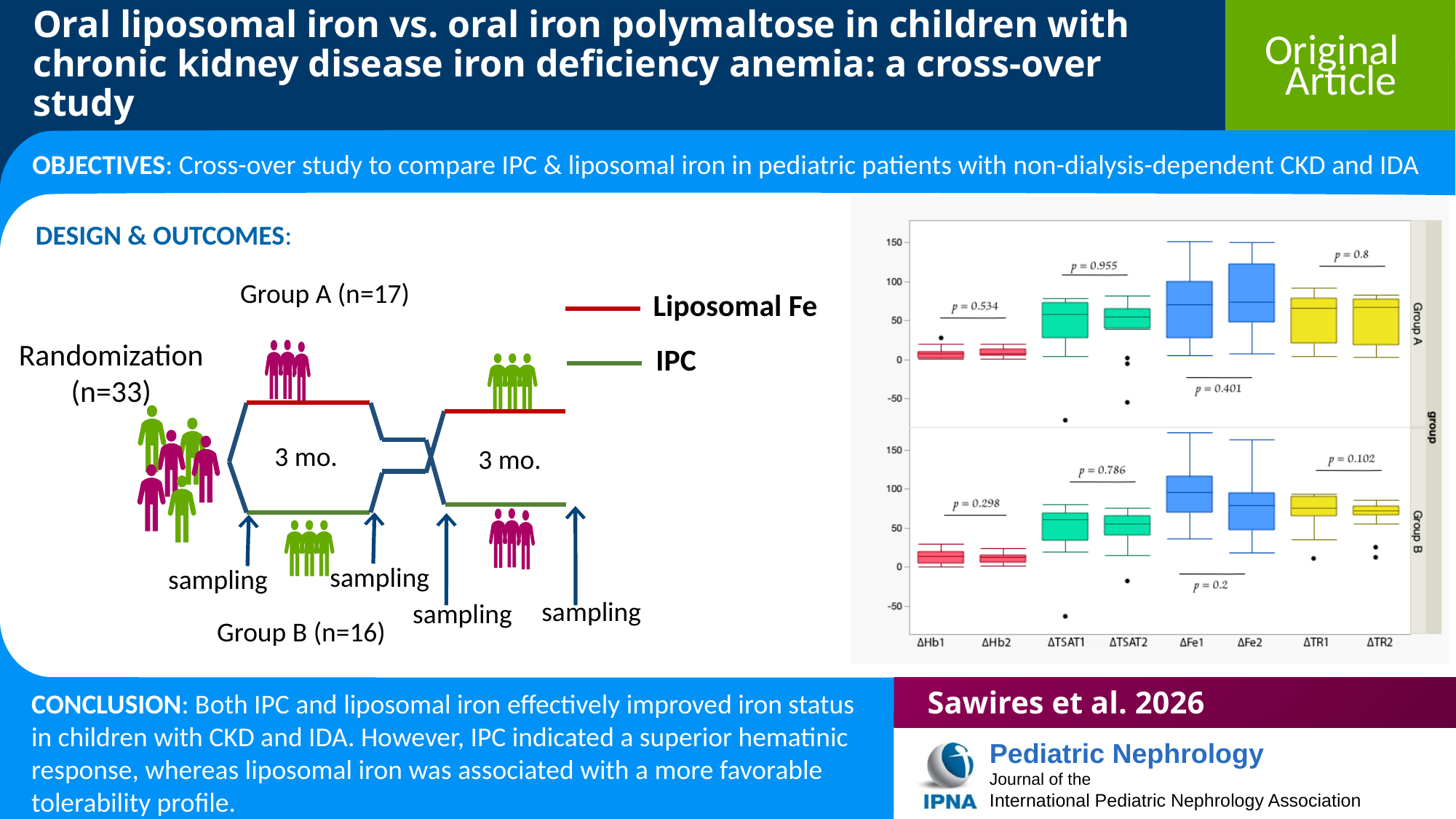

Oral liposomal iron vs. oral iron polymaltose in children with chronic kidney disease iron deficiency anemia: a cross-over study
OBJECTIVES: Cross-over study to compare IPC & liposomal iron in pediatric patients with non-dialysis-dependent CKD and IDA
DESIGN & OUTCOMES:
Group A (n=17)
Liposomal Fe
Randomization
(n=33)
IPC
3 mo.
3 mo.
sampling
sampling
sampling
sampling
Group B (n=16)
Sawires et al. 2026
CONCLUSION: Both IPC and liposomal iron effectively improved iron status in children with CKD and IDA. However, IPC indicated a superior hematinic response, whereas liposomal iron was associated with a more favorable tolerability profile.
