# Supplementary figures and images for: Oral liposomal iron vs. oral iron polymaltose in children with chronic kidney disease iron deficiency anemia: a cross-over study
Source: Pediatr Nephrol. 2026 Jan 15;41(6):1803–10. doi: 10.1007/s00467-025-07138-w (PMC13139247; doi:10.1007/s00467-025-07138-w)

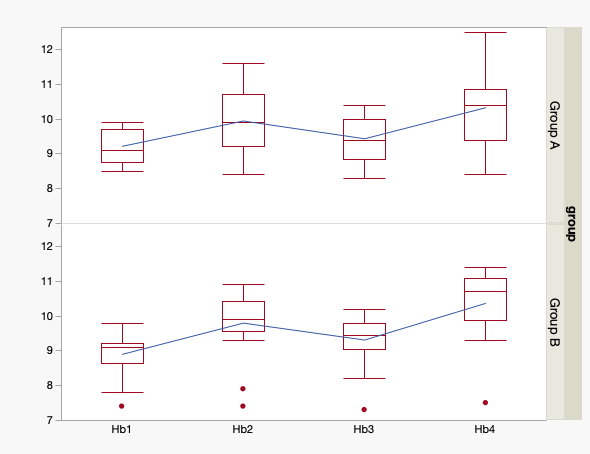

Supplement: Supplementary file 2 — Supplementary file 1 (PNG 21.2 KB ) [file 467_2025_7138_MOESM2_ESM.png]

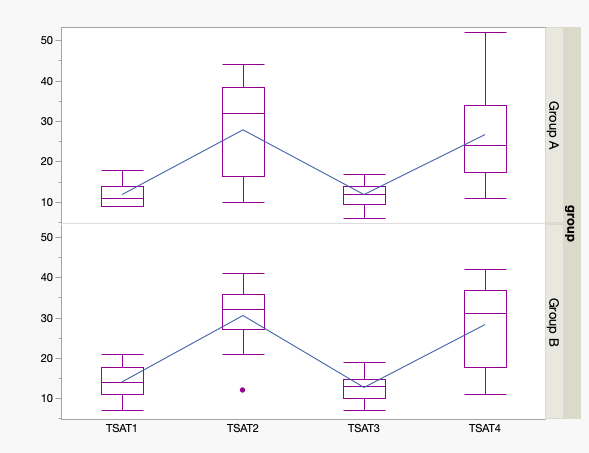

Supplement: Supplementary file 3 — Supplementary file 2 (PNG 26.0 KB) [file 467_2025_7138_MOESM3_ESM.png]
